# A Bimodal Pattern and Age-Related Growth of Intra-Annual Wood Cell Development of Chinese Fir in Subtropical China

**DOI:** 10.3389/fpls.2021.757438

**Published:** 2021-12-09

**Authors:** Yanyan Jiang, Xiongqing Zhang, Sophan Chhin, Jianguo Zhang

**Affiliations:** ^1^Key Laboratory of Tree Breeding and Cultivation of the National Forestry and Grassland Administration, Research Institute of Forestry, Chinese Academy of Forestry, Beijing, China; ^2^Collaborative Innovation Center of Sustainable Forestry in Southern China, Nanjing Forestry University, Nanjing, China; ^3^Division of Forestry and Natural Resources, West Virginia University, Morgantown, WV, United States

**Keywords:** age, wood cell development, microcores, Chinese fir, bimodal pattern

## Abstract

Age plays an important role in regulating the intra-annual changes in wood cell development. Investigating the effect of age on intra-annual wood cell development would help to understand cambial phenology and xylem formation dynamics of trees and predict the growth of trees accurately. Five intermediate trees in each stand (total of 5 stands) in five age groupings of Chinese fir (*Cunninghamia lanceolata* Hook.) plantations in subtropical China were monitored on micro-cores collected weekly or biweekly from January to December in 2019. We modeled the dynamics of wood cell development with a mixed effects model, analyzed the age effect on intra-annual wood cell development, and explored the contribution of rate and duration of wood cell development on intra-annual wood cell development. We found a bimodal pattern of wood cell development in all age classes, and no matter the date of peak or the maximal number of cells the bimodal patterns were similar in all age classes. In addition, compared with the older trees, the younger trees had the longest duration of wood cell development because of the later end of wood cell development and a larger number of wood cells. The younger trees had the faster growth rate than the older trees, but the date of the maximal growth rate in older trees was earlier than younger trees, which led to the production of more wood cells in the younger trees. Moreover, we found that the number of cells in wood cell formation was mostly affected by the rate (92%) rather than the duration (8%) of wood cell formation.

## Introduction

The intra-annual radial growth of trees can be monitored using dendrometers, pinning, and micro-coring (Seo et al., 2007; Drew and Downes, 2009). Compared with the other two methods, micro-coring could monitor cambial cell division and the dynamics of xylem cell formation directly, and combined with paraffin sections to show the details of wood cell development (cambial division, enlarging, wall-thickening, and maturation) (Gruber et al., 2010; Kalliokoski et al., 2011; Lenz et al., 2013). Moreover, wood microcores sampled by Trephor (PD2004A000324) had a small volume and produced little damage to trees, which could be collected continuously and help define the timing of wood cell development and calculate the rate of wood cell development (Zhai et al., 2012; Lupi et al., 2013; Cuny et al., 2014; Huang et al., 2014).

Older trees exhibit smaller tree rings than younger trees, resulting in a declining trend of ring-width series across the diameter from pith to bark (Panyushkina et al., 2003). Lundqvist et al. (2018) reported that the annual growth, fiber and wood properties of Norway spruce [*Picea abies* (L.) Karst.] change dynamically, particularly at young ages. Timing and duration of xylem formation differed between adult (50–80 years) and old (200–350 years) trees [*Larix decidua* Mill., *Pinus cembra* L. and *Picea abies* (L.) Karst.] which is typically 2–3 weeks shorter in both timing and duration of xylem formation in old trees and lead to reductions of 15–20% in the overall duration of xylem differentiation (Rossi et al., 2008). Zeng et al. (2017) also found that tree age played an important role in the timing and duration of growth in Chinese pine (*Pinus tabulaeformis* Carr.) and Qilian juniper (*Juniperus przewalskii* Kom.) in a semi-arid region of northwestern China. In the Tibetan Plateau, compared with old (162 ± 26 years) Smith fir [*Abies georgei* var. *smithii* (Viguie & Gaussen) W. C. Cheng & L. K. Fu], young (43 ± 4 years) Smith fir had an earlier onset of xylogenesis, a longer growing season and a higher growth rate, resulting in a higher number of xylem cells (Li et al., 2012).

Obviously, there is an evident effect of age on the number of cambial cell division and wood cell formation, timing, and duration of wood cell development and the rate of wood cell development. Furthermore, age also impacts wood structure and function (Rodriguez-Zaccaro et al., 2019), fibers (Lundqvist et al., 2018), and wood density (Sousa et al., 2016). However, most of these studies (Li et al., 2017; Ren et al., 2018; He et al., 2019; Castagneri et al., 2020; Huang et al., 2020; Tabakova et al., 2020; Versace et al., 2020; Zenga et al., 2020) focused on exploring the effect of exogenous factors on intra-annual wood cell development. Fewer studies (Rossi et al., 2008; Plavcova et al., 2013) investigated the effect of endogenous factors on intra-annual wood cell development although they had the non-negligible regulation on the intra-annual wood cell development, especially the effect of age on the intra-annual wood cell development. Investigating the effect of age on wood cell development provides improved understanding of cambial cell division and xylem cell formation dynamics of trees, and can in turn lead to a more accurate evaluation and prediction of radial growth.

In addition, the effect of age and altitude on wood formation and the relationship of “wood formation-climate” has been explored in temperature and boreal forests using the micro-sampling approach (Zhai et al., 2012; Huang et al., 2014). However, investigations of intra-annual wood formation in tropical and subtropical forests using the micro-sampling are still lacking (Morel et al., 2015; Bosio et al., 2016), as well as studies exploring the effect of age on wood cell development, despite there being ∼1.39 trillion trees in tropical and subtropical forests among 3.04 trillion trees globally (Crowther et al., 2015; Huang et al., 2018). Additionally, studies in tropical and subtropical forests indicated that seasonal cambium activity and xylem formation are species-specific (Morel et al., 2015; Bosio et al., 2016).

Moreover, studies in temperature and boreal forests showed a unimodal pattern of intra-annual xylem formation (Moser et al., 2009; Lupi et al., 2010; Zhang J. et al., 2018). However, studies in Mediterranean and tropical zones showed a bimodal pattern of intra-annual xylem formation: i.e., the cambial layer would be dormant during the growing season because of extreme environment and then resumed activity at the appropriate environment and led to two growing seasons in a year (Liphschitz et al., 1984; Venugopal and Krishnamurthy, 1987; Camarero et al., 2010). How about the dynamics of forests in subtropical climate zone? Is there a unimodal pattern or a bimodal pattern in the intra-annual wood cell development in subtropical forests?

Furthermore, a similar amount of xylem cells can be produced either with a faster growth rate over a shorter growing season, or with a slower growth rate maintained for a longer period (Deslauriers and Morin, 2005; Ren et al., 2015; Guo et al., 2019). Prislan et al. (2019) found that growing season length determines the radial growth of trees in beech (*Fagus sylvatica* L.) forest located in the Kamnik-Savinja Alps. In cold climate zones, the greatest growth increment was observed with cell productions lasting more than 70 days, and rate of cell production only marginally affected the number of cells. In addition, the analysis of Rossi et al. (2014) estimated the effects of the duration of cell production and rate of cell production at 86 days and 14%, respectively. In contrast, Ren et al. (2019) found that the number of xylem cells was mostly related to growth rate rather than duration of cell production. Exploring the effect of growth rate and growth duration could help to understand the dynamics of intra-annual radial growth.

Chinese fir is the most widely distributed with 9.9 million ha in China’s subtropical forests among the plantation species in China and has been increasingly planted in China due to its high economic and ecological values (Zhang et al., 2013; Wang et al., 2021). However, to our knowledge, no study has investigated the age dependent nature of intra-annual wood cell development of Chinese fir. The objectives of the study are to (I) identify the dynamics of intra-annual wood cell development (e.g., onset and end time of cambial cell division, duration and rate of wood formation) of Chinese fir in different age classes, (II) compare the differences in characteristics of wood cell development between different age classes, and (III) investigate the contribution of duration and rate of wood cell development to the intra-annual wood cell development. These results will be helpful for evaluating the carbon reserve of Chinese fir stands at a higher temporal resolution.

## Materials and Methods

### Study Area and Stands

The experimental site is located in Shanxia forest farm, situated in Jiangxi province in southern China (Supplementary Figure 1). It has a mean annual temperature of 17.9°C and a mean annual precipitation of 2047.5 mm. In November of 2018, we chose five stands with different age classes growing in similar site conditions in the forest farm: trees planted in 2012, 2006, 2000, 1993, and 1969. In each stand, a permanent plot with an area of 20 m × 30 m was established. All trees were tagged in each plot, and their heights, diameter at breast height and crown width were measured in November of 2018.

According to their diameters at breast height, five intermediate trees with upright and injury-free stems were selected for sampling in each plot (Table 1). Trees with partially dead crowns or evident damage were avoided.

**TABLE 1:** Age and size of the sampled trees.

Planting year	Age (years)	Age classes	N (trees/ha)	DBH (cm)	H (m)	CA (m^2^)
2012	8	Young	3,433	8.6 (0.46)	6.7 (0.21)	4.5 (1.00)
2006	14	Middle	2,800	12.6 (0.43)	12.8 (1.27)	5.0 (1.57)
2000	20	Near-mature	766	16.7 (0.31)	13.6 (0.37)	10.3 (2.81)
1993	27	Mature	766	21.0 (0.39)	13.6 (0.39)	8.9 (1.19)
1969	51	Over-mature	583	22.3 (0.74)	16.7 (0.98)	13.7 (1.58)

*The values in brackets are standard deviation values.*

*N, represents the stand density. DBH, represents the average diameter at breast height of five sampled trees in each stand. H, represents the average height of five sampled trees in each stand. CA, represents the average area of tree crown of five sampled trees in each stand.*

### Sampling Design and Laboratory Preparation

Tree-ring formation was studied from January to December 2019. Wood microcores (15 mm long, 2 mm diameter) with phloem, cambial and xylem were collected with a sampling frequency of every 7–10 days on the stem from 30 cm below to 30 cm above breast height (1.3 m) at the same aspect using Trephor microcore sampling tool (Rossi et al., 2006a; Supplementary Figures 2A,B). Wood samples were always taken at least 5 cm apart to avoid the influence from adjacent cores (Deslauriers et al., 2003). About 825 microcores were collected from the 25 trees weekly or biweekly over the year. The collected microcores were fixed in formalin-ethanol-acetic acid solution (Supplementary Figure 2C) and stored at 5°C to avoid tissue deterioration (Ren et al., 2019). The microcores were dehydrated with successive immersions in ethanol, D-limonene and paraffin, then embedded in paraffin and transverse sections of 8–10 μm thickness were cut with a rotary microtome (Rossi et al., 2006a).

### Determination of Xylem Development

Sections were stained with safranin and fast green and observed under the visible and polarized light at 400–500 × magnifications to distinguish the enlarging cells and wall-thickening cells (Rossi et al., 2008; Huang et al., 2018). In cross-section, cambial cells were characterized by thin cell wall and small radial diameters (Zhang S. et al., 2018). The enlarging cell was still composed of the thin primary wall but with radial diameter at least twice that of a cambial cell (Rossi et al., 2008). Because of the arrangement of the cellulose microfibrils, the developing secondary walls glistened when observed under polarized light, and no glistening was observed in enlargement zones (Supplementary Figure 3B; Lupi et al., 2010). Given that safranin can react with the lignin, we also detected the progress of cell wall lignification, as indicated by a color change from green to pink. Furthermore, a homogenous pink color over the whole cell indicates the end of lignification and the maturity of a tracheid (Gricar et al., 2005). Three rows of cambial and xylem cells in each slice were selected to count the number of cambium cells, enlarging cells, wall-thickening cells and mature cells (Rossi et al., 2006b; Huang et al., 2014). The total number of wood cells was calculated as the sum of cambium, enlarging, wall-thickening and mature cells.

The number of xylem cells in the three rows per tree were averaged and used to assess onset, end, and duration of xylem growth. In spring, when at least one row of cells was observed in the enlarging phase, the wood cell development was considered to begin. In the late summer, when no new enlarging cells were observed, and no cells were observed in wall thickening and lignification stages, the wood cell development was considered accomplished. The assessment of the onset, end, and duration of wood cell development in our study was based on the enlargement phase according to Guo et al. (2020). The appearance of the first enlarging cell and last enlarging cell of each tree were identified as the onset and end of wood cell development, respectively. The duration of wood cell development was assessed according to the period between the appearance of the first and last enlarging cell.

### Statistical Analyses

A S-shaped curve, Gompertz function, has been widely used in modeling the increase in wood cell number (Rossi et al., 2003; Zhang J. et al., 2018; Zhang S. et al., 2018), which was defined as follows:


(1) Y=A⁢exp⁢(-exp⁢(β-kt))

where *Y* is the total number of cells at the time t; *A* is the asymptote parameter representing the final number of cells reached at the end of the growing season; β is the time axis placement parameter which reflects the choice of the origin time; and *k* is the growth-rate parameter that determines the spread of the curve along the time axis. These parameters were estimated using the package “nlme” in the R software (Pinheiro et al., 2017; R Core Team, 2018), which involves the fitting of nonlinear mixed-effects models by maximizing the restricted log-likelihood. In the model, tree was considered as the random factor, which was added to the asymptote parameter *A*.

From the estimated parameters of the Gompertz function, the date of the inflection point (*t*_*p*_) and the corresponding maximal growth rate (*r*_*max*_) were computed:


(2) $${\text{t}}_{\text{p}}=\beta /k$$

(3) $${\text{r}}_{\text{max}}=\text{kA}/e$$

where *e* is the Euler number, which approximately equals to 2.72 in our study.

The daily growth rate (*r*) of wood cell formation was calculated as the slope of the modeled S-shape growth curve. In addition, the average growth rate (*r*_*m*_)—computed between *t*5 and *t*95—was completed:


(4) $${\text{r}}_{\text{m}}=\frac{0.95\,A-0.05\,\text{A}}{{\text{t}}_{95}-{\text{t}}_{5}}\approx \frac{9}{40\,{\text{er}}_{\text{max}}}$$

where *t*_5_ represents the date at which 5% of the wood cell development is reached, and *t*_95_ represents the date at which 95% of the wood cell development is achieved.

For examining the effects of rate and duration on wood cell development, the following linear regression was used (Nally, 2002):


(5) $$\text{Y}=\alpha_{0}+\alpha_{1}\,{\text{x}}_{1}+\alpha_{2}\,{\text{x}}_{2}$$

where *Y* is the total number of cells of xylem cell formation in the year, *α_0_* is the intercept, *α_1_* and *α_2_* are the parameters, *x*_1_ is the rate of xylem cell formation, and *x*_2_ is the duration of xylem cell formation.

In addition, the R package “relaimpo” was used to calculate the contribution of the rate and duration of xylem cell formation more precisely (Gromping, 2006). Dynamics of cambial cells, enlarging, wall-thickening and mature cells were modeled with the “smooth function” in R to show original dynamics.

For each age class, the onset, end, and duration of wood cell development were computed in days of the year. For exploring the effects of age on the number of cells, onset, end, and duration of wood cell formation, characteristics of rate of wood cell formation, analysis of variance (ANOVA) was performed in the study.

## Results

### The Pattern of Wood Cell Development

**FIGURE 1:**
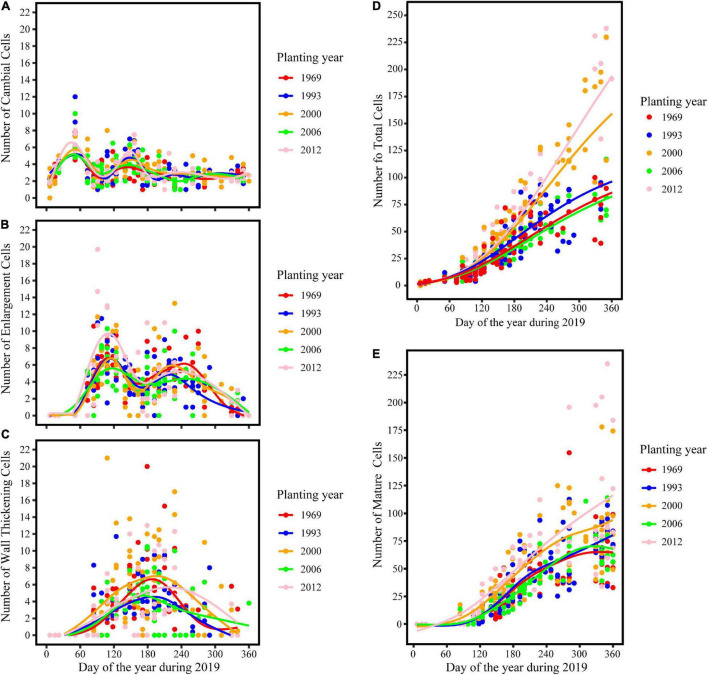
Dynamics of different years of plantation establishment and aspects of wood cell development. **(A)** Cambial cells; **(B)** enlarging cells; **(C)** wall thickening cells; **(D)** total number of cells; **(E)** mature cells.

The intra-annual dynamics of wood cell development could be fitted well by the Gompertz function, with *R*^2^ = 0.92, *R*^2^ = 0.89, *R*^2^ = 0.92, *R*^2^ = 0.82, *R*^2^ = 0.76 for the young, middle, near-mature, mature, and over-mature stands, respectively. Furthermore, all the parameters were significant at the level of 0.05 (Table 2). All trees in different age classes showed the similar trend of wood cell development but with a different timing of the inflection point and different number of cells (Table 3). A bimodal pattern was detected in number of cambial cells and number of enlarging cells (Figures 1A,B). A bell-shaped curve was found in the number of wall-thickening (Figure 1C). The dynamics of the number of mature cells and total number of cells was S-shape (Figures 1D,E).

**TABLE 2:** Parameter estimates and their standard errors of the model for the number of cells produced during wood cell development of Chinese fir plantations using the Gompertz equation.

Planting year	Parameter	Estimates	Std.error	df	*T*-value	*R* ^2^
2012	A	358.5	87.720	52	4.09	0.92
	k	1.68	0.040	52	39.68	
	t	0.006	0.001	52	9.07	
2006	A	116.4	20.56	73	5.66	0.89
	k	1.46	0.040	73	34.26	
	t	0.007	0.001	73	9.10	
2000	A	243.4	41.51	78	5.86	0.92
	k	1.67	0.050	78	32.89	
	t	0.007	0.0008	78	8.88	
1993	A	123.4	33.92	82	3.64	0.82
	k	1.49	0.070	82	20.10	
	t	0.008	0.001	82	5.59	
1969	A	94.7	25.780	84	3.68	0.76
	k	1.38	0.080	84	17.49	
	t	0.008	0.0013	84	5.91	

*All p-values < 0.001.*

*Std.error, represents standard error. A, represents the asymptote parameter that determines the final number of cells reached at the end of the growing season. k, represents the growth-rate parameter that determines the spread of the curve along the time axis. t, represents the day of the year. df, represents degree of freedom.*

**TABLE 3:** Inflection points of different cell stages.

Planting year		Cambium				Enlargement				Wall-thickening	Wall-thickening
		Ft	Fb	St	Sb	Ft	Fb	St	Sb	t	b
DOY	2012	51 (0)	108 (0)	151.2 (3.9)	189.4 (5.2)	108 (0)	169.4 (5.9)	236 (4)	335.4 (9.5)	191 (6.8)	321.8 (8.8)
	2006	51 (0)	101.2 (9.4)	149.6 (3.2)	186.8 (6.4)	106 (4)	163 (0)	240 (7.5)	337.8 (4.4)	181.6 (5.2)	327.6 (9.3)
	2000	51 (0)	91.6 (4.6)	152.6 (6.1)	189.4 (5.2)	110 (4)	166.4 (6.1)	234 (4.9)	335.6 (5.4)	192.6 (7.7)	306.8 (8.4)
	1993	51 (0)	95.8 (8.0)	149.6 (3.2)	186.8 (6.4)	114 (4.9)	164.6 (3.2)	228 (8.9)	337.6 (7.9)	166.2 (3.9)	306.8 (8.4)
	1969	51 (0)	94.4 (8.1)	151 (7.9)	192 (0)	108 (6.3)	164.8 (5.7)	234 (4.9)	335.6 (5.4)	169.4 (7.8)	325.4 (7.2)
NC	2012	7 (0.9)	2.7 (0.5)	6.3 (1.0)	2.4 (0.3)	12.7 (1.5)	0.8 (0.9)	6.3 (0)	1.1 (0.8)	8.0 (4.1)	1.2 (1.7)
	2006	6.5 (2.1)	1.8 (0.4)	5 (1)	2.2 (0.3)	8.3 (1.1)	3.4 (0.5)	6 (2.4)	2.0 (0.5)	7.8 (2.0)	0 (0)
	2000	7.1 (0.8)	1.7 (0.7)	5.8 (0.4)	2.7 (0.6)	7.2 (2.0)	1.5 (1.3)	9.8 (3.5)	0.3 (0.3)	10.7 (1.8)	0.8 (1.1)
	1993	9 (2.5)	1.9 (0.5)	6 (0.8)	1.9 (0.3)	9 (1.1)	3.4 (0.5)	5.8 (0.7)	1 (0.8)	7.8 (0.8)	0 (0)
	1969	6.6 (1.5)	1.5 (0.8)	4.6 (0.5)	2.2 (0.5)	7.8 (1.1)	2.6 (1)	4.6 (0.2)	0 (0.2)	8.2 (5.6)	0 (0)

*The values in brackets are standard deviation values.*

*Ft, represents the first maximum. Fb, represents the first minimum. St, represents the second maximum. Sb, represents the second minimum. t, represents the maximum. b, represents the minimum. DOY, represents the day of the year in 2019. NC, represents the number of cells.*

The maximum number of cambial cells appeared in middle of February, and then the number of cambial cells decreased. By the middle of April, the number of cambial cells reached the minimum value. By the end of May and start of June, the maximum number of cambium cells appeared again, but the value was smaller than the first maximum value. In the middle of July, the number of cambial cells reduced to 2–3 cells and kept a stable value. In contrast to the cambial cells, the number of enlarging cells increased from the middle to the end of February. The first maximum number of cells appeared around the middle of April, and reduced to the minimum number by the middle of June. The second maximum number of cells appeared in the middle to end of August, but the value was smaller than the former value. By the end of November, the number of cells reduced to 0–3 cells. The date of the maximum number of wall-thickening cells was similar to the date of the second minimum number of cambial cells and the first minimum number of enlarging cells. After that, the number of wall-thickening cells reduced. By the middle of November, the number of wall-thickening cells reduced to a minimum value, which was around 0–3 cells (Figures 1A–C and Table 3).

The dynamics of mature cells in five classes were S-shape curve (Figure 1E), and had a significant age effect on the time of the appearance of the first mature cell and the number of mature cells: i.e., the first mature cell appeared earliest in the trees at the young stand, followed by trees of the near-mature stand, mature stand, and over-mature stand. Furthermore, trees in the young stand had the highest number of cells (204.5 cells), followed by trees in the near-mature stand (155.8 cells), mature stand (83 cells), and over-mature stand (63.2 cells) (Figure 1E). Compared with other age classes, the middle-aged stand had the largest stand density (Table 1), and the effect of stand density on radial growth was larger than the effect of age on radial growth; consequently, the growth curve of the middle-aged stand was similar with the mature stand and over-mature stand (Figure 1E and Table 3).

### Rate of Wood Cell Development

**FIGURE 2:**
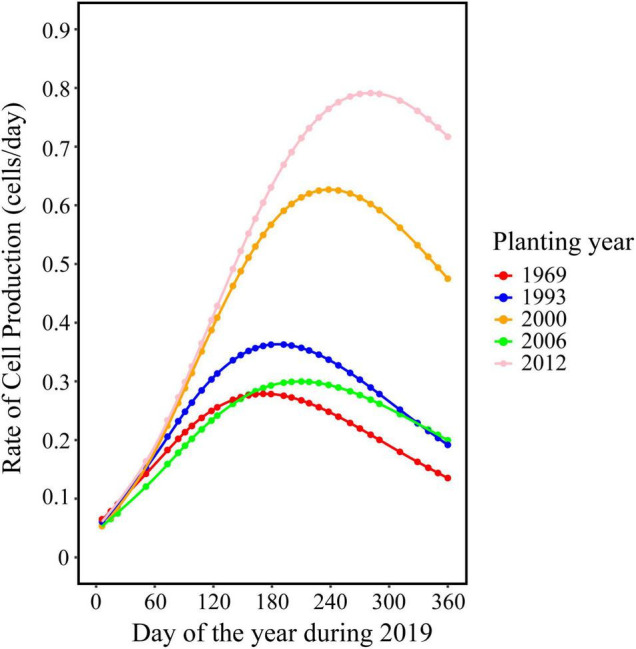
Rate of wood cell development for stands with different planting years.

The trend in the rate of wood cell development was similar between the five age classes with a significant effect of age on the value of maximum and the date of maximum (Figure 2). Trees in young stand had the highest rate of wood cell formation, with a maximal rate was 0.83 cells/day and an average rate of 0.51 cells/day, followed by trees in near-mature stand (the maximal and average rate was 0.65 and 0.4, respectively), mature stand (0.34 and 0.21, respectively), middle-aged stand (0.31 and 0.19, respectively), and over-mature stand (0.27 and 0.17, respectively). Moreover, the date of the maximal rate was different in five age classes: trees in over-mature stand appeared earliest, followed by trees in mature stand stage, near-mature stand stage, middle-aged stand stage, and young stand (Table 4).

**TABLE 4:** Characteristics of the rate of wood cell development.

Planting year	Sample size	DOY	r-max	r-m	Onset	End	Duration
2012	59	264.7	0.83	0.51	75.2 (4.4)	318 (20.7)	242.8 (24.7)
2006	80	200.4	0.31	0.19	76.6 (7.2)	315.8 (18.7)	239.2 (17.6)
2000	85	229.4	0.65	0.4	77.4 (5.6)	306.2 (21.6)	228.8 (22.5)
1993	89	198.6	0.34	0.21	77.4 (5.4)	298.4 (18.8)	221 (13.8)
1969	91	178.5	0.27	0.17	78.8 (7.4)	290 (22.4)	211.2 (26.2)

*The values in brackets are standard deviation.*

*DOY, represents the day of the year in 2019. r-max, represents the maximal rate of cell production. r-m, represents the average rate of cell production.*

### Duration of Wood Cell Development

**FIGURE 3:**
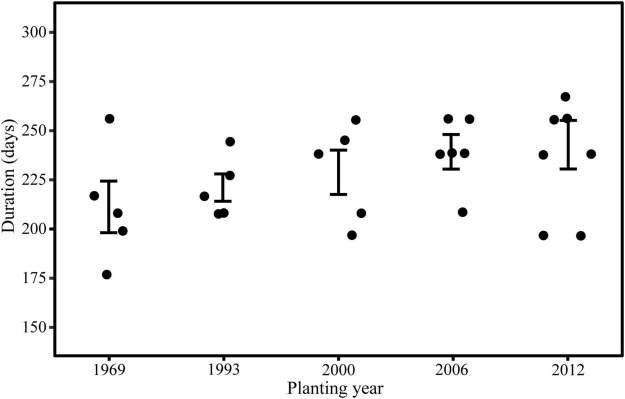
Duration of wood cell development compared between stands of different planting years.

Trees in young stand had the longest duration of wood cell development, followed by the middle-aged stand, near-mature stand, mature stand, and over-mature stand (Figure 3). The onsets of wood cell development were similar in the five age classes. All trees started to grow in 77^th^ day of the year. However, the ends of wood cell development were different in the five age classes: trees in young stand ended at 318 ± 20.7 days of the year, trees in middle-aged stand ended at 315.8 ± 18.7 days of the year, trees in near-mature stand ended at 306.2 ± 21.6 days of the year, trees in mature stand ended at 298.4 ± 18.8 days of the year, and trees in over-mature stand ended at 290 ± 22.4 days of the year, which in turn resulted in different durations of wood cell development (Table 4).

### The Effect of Age on Wood Cell Development

According to the results of ANOVA, the number of wood cell development, the end and duration of wood cell development, the date of the maximal rate of wood cell development, the maximal and average rate of wood cell development had a significant age effect (*P* ≤ 0.05), while the onset of wood cell development was not significant (*p* > 0.05, Table 5).

**TABLE 5:** The summary results of ANOVA.

	df	Sum-Sq	Mean-Sq	*F*-value	*p*-value
Number	1	9704.00	9704.00	10.58	0.004
Onset	1	29.60	29.60	0.73	0.401
End	1	2504.00	2504.10	5.35	0.030
Duration	1	3078.00	3077.80	5.99	0.023
t-p	1	23912.00	23912.00	41.56	<0.001
r-max	1	0.30	0.30	15.11	<0.001
r-m	1	0.15	0.15	15.15	<0.001

*Where p-value ≤ 0.05 indicates significant differences.*

*t-p, represents the time of the maximal rate of xylem cell formation. r-max, represents the maximal rate of xylem cell formation. r-m, represents the average rate of xylem cell formation. Df, represents the degree of freedom of one-way analysis of variance. Sum-Sq, represents sum of square. Mean-Sq, represents mean of square.*

### The Contribution of Duration and Rate on Wood Cell Development

The rate of wood cell development had a significant effect on wood cell development (*p* ≤ 0.05), while the duration of wood cell development did not have a significant effect on wood cell development (*p* = 0.067 (Table 6). Moreover, we analyzed the contribution of duration and rate to wood cell development using R package “relaimpo,” and found that 92% of wood cell development was explained by the rate of wood cell development, while 8% of wood cell development was explained by duration. Thus, the number of wood cell development was much more sensitive to the rate than the duration of wood cell development.

**TABLE 6:** Parameter estimates of wood cell development linear model against rate and duration.

	value	*T*-value	*p*-value
Intercept	–56.36	–2.58	0.019
Rate	397.28	13.6	<0.001
Duration	0.19	1.95	0.067

*p-value ≤ 0.05 indicates significant differences.*

## Discussion

### A Bimodal Pattern of Wood Cell Development

We found that there was a bimodal pattern in the dynamics of the cambial and enlarging phases and divided the wood cell development into two periods, which is similar to the pattern of conifer species (*Juniperus thurifera*, *Pinus halepensis*, and *Pinus sylvestris*) endemic to the western Mediterranean Basin (Spain, Morocco, France and Algeria) (Camarero et al., 2010). In their context, the bimodal pattern of xylem formation was characterized by spring and autumn precipitation; furthermore, in order to reduce the water consumption during the drought condition, the cambial cells would be in dormancy, and could resume the cell division when the moisture levels are suitable (Camarero et al., 2010).

Cambium division and post-cambial growth are strictly connected (Rossi et al., 2003, 2006b): once the enlarging phase started, the number of cambial cells will be reduced, and thus a bimodal pattern of cambial zone resulted in a bimodal pattern of the enlarging phase.

### The Effect of Age on Characteristics of Wood Cell Development

As reported by Rossi et al. (2008), our results showed that trees in five age classes had a similar onset but different timing of the end of wood cell development (Table 4). In the early growth phase, the onset of radial growth was potentially controlled by the exogenous factors such as temperature and precipitation (Piper et al., 2005; Rossi et al., 2011; Ren et al., 2018). However, in contrast to the early growth, the effect of exogenous factors is likely reduced in the late stages of growth and the late growth was mainly controlled by endogenous factors (Luo et al., 2018; Wang et al., 2020), which led to trees in five age classes having the different timing for the end of wood cell development.

Our results showed that trees in the young stand produced the highest number of cells while trees in over-mature stand produced a lower number of cells (Figure 1D), which supported the view that intra-annual radial growth changed significantly with tree age (Melesse and Zewotir, 2018). Except at the later end of wood cell development, younger trees produced a higher number of cells along the radial direction in the stem, which may also lead to younger trees having the longer duration of wood cell development (Gricar et al., 2005; Li et al., 2012). In addition, the rate of radial growth had a significant age effect: younger trees had a faster growth rate, whereas older trees had a slower growth rate, which was also found by Li et al. (2012). Compared with younger trees, older trees may have a higher hydraulic resistance and lower photosynthetic rates (Mérian and Lebourgeois, 2011), which might reduce the growth rate of older trees.

Furthermore, we also found that the timing of the maximal growth rate revealed a strong age effect, with older trees experienced the maximal growth rate earlier, whereas younger trees experienced the maximal growth rate later. This difference may be because the trees in different age classes may have a different sensitivity of climate (Rozas, 2005): i.e., younger trees had a longer xylogenetic activity and produced a dilution of the climatic signal over a longer period, and thus reduced the response level to climate (Carrer and Urbinati, 2004).

### Contribution of Duration and Rate

Our results showed that similar duration but significantly different rate of wood cell development led to significantly different number of xylem cells in the year. This was confirmed by our analysis that 92% of the wood cell formation was contributed by the rate of wood cell formation, while 8% of the wood cell formation was contributed by the duration of wood cell development. In addition, the intra-annual wood cell development in our study was mainly related to the growth rate rather than duration of radial growth, which is different from studies in boreal forests (Griffis et al., 2003; Prislan et al., 2019), but was observed by studies in the temperature forests and north-eastern Tibetan Plateau area (Ren et al., 2015, 2019; Zhang J. et al., 2018).

## Conclusion

Our study indicated there was an age-related signal in wood cell development of Chinese fir with respect to the number of wood cell development, the end and duration of wood cell development, the date of the maximal rate of wood cell development, the maximal and average rate of wood cell development. The similar bimodal pattern in all age classes might be due to exposure to a similar set of exogenous factors which should be examined in detail in future studies. The cell number of wood cell development was related to growth rate and duration, but was mainly dependent on the former (92%) compared to the later in our study. These results have contributed to improving understanding of the pattern of cambial phenology and xylem formation dynamics and evaluating the carbon reserves of Chinese fir stands in subtropical China at a higher temporal resolution.

## Data Availability Statement

The original contributions presented in the study are included in the article/Supplementary Material, further inquiries can be directed to the corresponding author/s.

## Author Contributions

YJ did the main experiments, analyzed the data, and wrote the draft. XZ conceived and funded the work, helped with the data analysis, wrote, and improved the draft. SC and JZ reviewed and improved the draft. All authors contributed to the article and approved the submitted version.

## Conflict of Interest

The authors declare that the research was conducted in the absence of any commercial or financial relationships that could be construed as a potential conflict of interest.

## Publisher’s Note

All claims expressed in this article are solely those of the authors and do not necessarily represent those of their affiliated organizations, or those of the publisher, the editors and the reviewers. Any product that may be evaluated in this article, or claim that may be made by its manufacturer, is not guaranteed or endorsed by the publisher.
