# Supplementary material for: A Bimodal Pattern and Age-Related Growth of Intra-Annual Wood Cell Development of Chinese Fir in Subtropical China
Source: Front Plant Sci. 2021 Dec 9;12:757438. doi: 10.3389/fpls.2021.757438 (PMC8695768; doi:10.3389/fpls.2021.757438)
Supplement: Supplementary file 1 [file Data_Sheet_1.docx]

Supplementary Figures


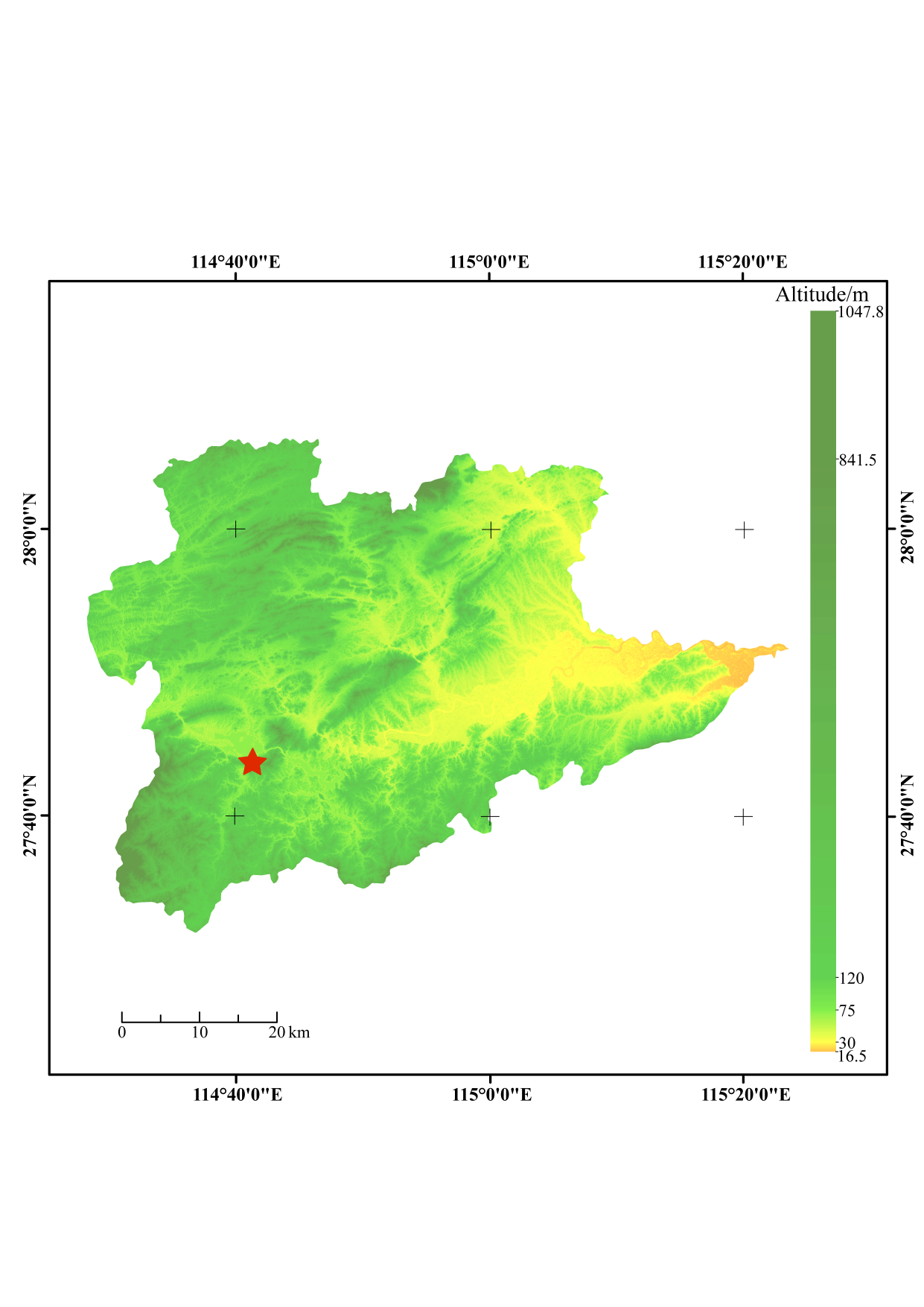


**Supplementary Figure 1.** Location of study region in Jiangxi Province. Red pentagram represents the study site.


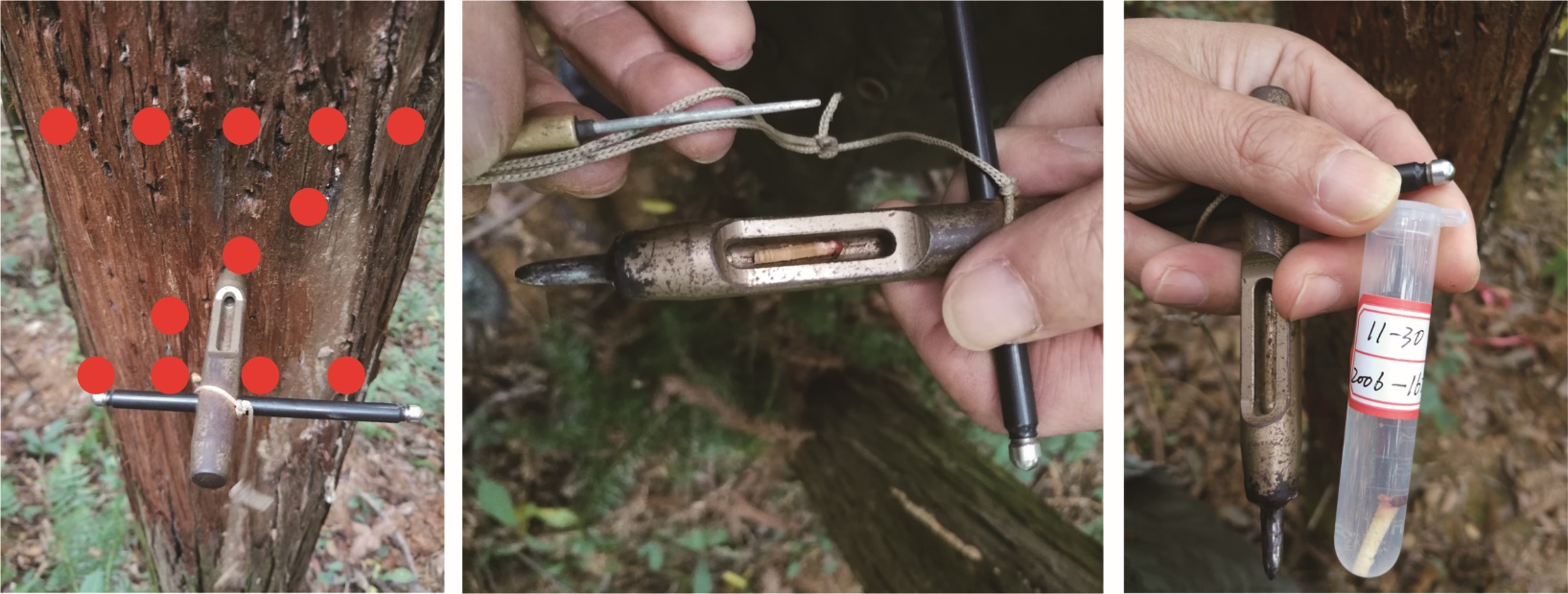


**(A)**

**(C)**

**(B)**

**Supplementary Figure 2.** The micro-sampling process in the field. **(A)**: the sampling site on the tree stem following the Z-shape; **(B)**: the sampled wood core which is 15 mm long and 2 mm in diameter; **(C)**: wood core fixed in FAA (formalin-ethanol-acetic acid solution).


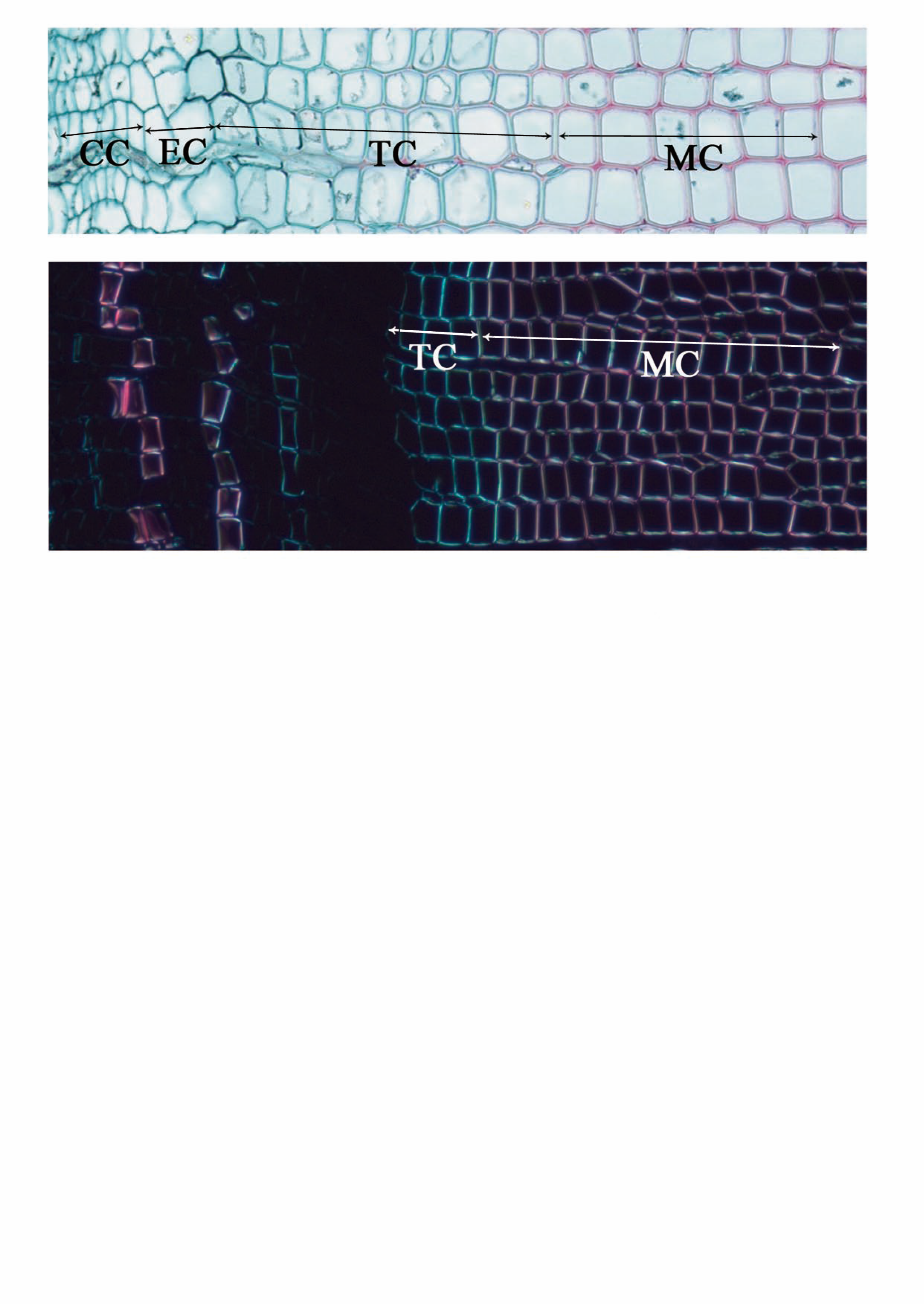


**(B)**

**(A)**

**Supplementary Figure 3.** Phases of wood cell development in Chinese fir. **(A)**: section stained with safranin and fast green. **(B)**: section with polarized light. CC represents the cambial cells, EC represents the enlarging cells, TC represents the wall-thickening cells, MC represents the mature cells.
